# Diagnosis and Orthodontic Treatment of Obstructive Sleep Apnea Syndrome Children—A Systematic Review

**DOI:** 10.3390/diagnostics14030289

**Published:** 2024-01-29

**Authors:** Kenan Ferati, Arberesha Bexheti-Ferati, Andrea Palermo, Carmen Pezzolla, Irma Trilli, Roberta Sardano, Giulia Latini, Alessio Danilo Inchingolo, Angelo Michele Inchingolo, Giuseppina Malcangi, Francesco Inchingolo, Gianna Dipalma, Antonio Mancini

**Affiliations:** 1 Faculty of Medicine, University of Tetovo, 1220 Tetovo, North Macedonia; kenan.ferati@unite.edu.mk (K.F.); arberesha.ferati@unite.edu.mk (A.B.-F.); 2 College of Medicine and Dentistry, Birmingham B4 6BN, UK; andrea.palermo2004@libero.it; 3 Interdisciplinary Department of Medicine, University of Bari “Aldo Moro”, 70124 Bari, Italy; c.pezzolla3@studenti.uniba.it (C.P.); trilliirma@gmail.com (I.T.); robertasardano@gmail.com (R.S.); dr.giulialatini@gmail.com (G.L.); ad.inchingolo@libero.it (A.D.I.); angeloinchingolo@gmail.com (A.M.I.); giannadipalma@tiscali.it (G.D.); dr.antoniomancini@gmail.com (A.M.)

**Keywords:** OSA(S), orthodontics, orthodontic treatment, sleep apnea, OSAS disease

## Abstract

Obstructive sleep apnea syndrome (OSAS) is a respiratory illness that is associated with recurrent episodes of either partial or full obstruction of the upper airways, or apnea, among other sleep disorders. This study aims to analyze, through a literature review, whether orthodontic treatment can be a good treatment strategy for this type of disorder. We performed a database search on Scopus, Web of Science, and Pubmed with the keywords OSA(S) and orthodontics to select the papers under evaluation. The criteria for inclusion were articles related to OSA(S) children undergoing an orthodontic treatment and clinical studies or case series, excluding systematic reviews, narrative reviews, meta-analyses, adult studies, animal models, and in vitro studies. The screening phase ended with the selection of 16 publications for this work. RME, or rapid maxillary expansion, turned out to be the preferred orthodontic treatment in cases of pediatric OSAS. The goal of this orthodontic procedure is to increase the hard palate’s transverse diameter by reopening the mid-palatal suture. Children with maxillary contraction and dental malocclusion typically undergo such a procedure and have excellent results. However, OSAS is a multifactorial disorder; it does not seem related to the morphology of the oral cavity, and therefore, it is not always possible to cope with this problem exclusively through orthodontic treatment.

## 1. Introduction

Sleep is a periodic, natural, biological occurrence that includes the loss of awareness, a reduction in or partial cessation of nerve center functioning, and a slowdown in the performance of certain bodily activities like breathing, circulation, and metabolism [1].

It turns out that sleep is essential to human existence; just consider that each person sleeps for roughly one-third of their lifetime [2,3]. Because it is intimately linked to the preservation of brain metabolism, the health of the rest of the cardiovascular system, and the equilibrium of glucose metabolism, it plays a significant role in preserving psychophysical balance [4,5,6,7,8,9,10,11,12]. Therefore, it is easy to see how sleep abnormalities could impact a person’s psychological and mental health [13,14,15]. Speaking of a child makes the situation considerably more significant [16,17,18]. Consider that a newborn’s first few months of life are spent sleeping for 70–80% of the day [19]. The first week of life is estimated to require 16–17 h of sleep, followed by 15 h at around 6 months, 14 h at roughly 1 year, 13 h at around 2 years, and 12 h at around 3 years [20]. After the age of six, children typically sleep for nine hours, ten hours, and eight hours during puberty [21,22,23,24,25,26,27,28,29,30,31,32]. The young sleep more because sleep serves multiple purposes, including boosting immune system strength, allowing the brain to “cleanse” waste toxins produced during wakefulness, consolidating memory and learning, promoting the release of growth hormones, and promoting brain development (especially REM sleep) [19,33,34]. Because of this, it is important to try to catch sleep disturbances in children early, on as they have a significant impact on their health. Obstructive sleep apnea syndrome (OSAS) is a respiratory illness that is associated with recurrent episodes of either the partial or full obstruction of the upper airways, or apnea, among other sleep disorders [35,36,37]. They can be of two types: peripheral, caused by a mechanical obstruction of the airways, or central, caused by a disruption in the neurological system’s capacity to stimulate the breathing muscles [38,39]. Just consider how common this is: depending on the case studies and polysomnographic criteria employed, the frequency of obstructive sleep apnea syndrome (OSAS) in children ranges from 0.69% to 5.7% [40,41].

### 1.1. Risk Factors

The main risk factors for the development of OSAS in children are adenotonsillar hypertrophy, obesity, craniofacial abnormalities, neuromuscular disorders, and hypercapnia [42] (Table 1).

#### 1.1.1. Hypertrophy of Adenotonsillary

The most typical risk factor for the onset of OSAS is adenotonsillary hypertrophy. Adenotonsillary hypertrophy in children peaks in occurrence between the ages of 2 and 6 [43,44,45]. Within the same age range, the transverse section and volume of the upper airways are smaller, and the adenotonsillary volume-to-airway ratio is favorable to the former [46,47,48]. After six years, the ratio reverses, and the airway’s transverse volume/section increases, but this is typically not accompanied by an increase in tonsillar or adenoid volume (the latter often tends to decline until normalization is achieved) [11,49,50,51]. Significant obstruction of the upper respiratory tract can occur when the tonsils and adenoids increase their encumbrance in the coan space and the hypopharynx, respectively [52,53,54]. However, significant adenotonsillary hypertrophy must still be linked to an upper airway-relative hypotonia for OSAS to manifest [55,56]. There is no obvious correlation between the adenoids and tonsils’ sizes and the severity of OSAS, and not all children with notable adenotonsillary hypertrophy have OSAS [57,58,59].

The condition of mono- or bilateral cleft lip and palate also affects the volume of the airway and nasopharyngeal space. A study by Kiaee showed a significant reduction in oropharyngeal and total volume in 30 patients aged 9 to 12 years with unilateral cleft lip and palate compared with 30 age- and sex-matched controls (*p* < 0.05) [60]. 

#### 1.1.2. Obesity

Owing to the substantial quantities of adipose tissue that are accumulated at the level of the ribs, upper airways, and abdomen, obesity results in a reduction in minute ventilation, as well as static and dynamic lung volumes and capacities [61,62]. The close relationship between respiratory disorders in sleep and obesity and, specifically, between OSAS and obesity can be explained by the combination of changes in respiratory function brought on by obesity and those physiologically determined by sleep [61]. OSAS is far more common in obese subjects than in the general population [63,64,65,66]. Prevalence values range from 14% to 78% [67]. These differences result from how obesity and OSAS are defined by different authors [68,69,70,71,72,73]. Studies have shown that the degree of obesity and the severity of OSAS are correlated and that adenotonsillary hypertrophy is more common as a risk factor in obese subjects than in the non-obese population with OSAS [18,74,75].

#### 1.1.3. Craniofacial Syndromes

The obstruction and appearance of OSAS are linked to primitive skeletal anatomical modifications of the upper airways, which are connected to syndromes of the craniofacial region [76,77]. Anatomical abnormalities in the upper airways associated with hypotonia and, in some cases, obesity may account for the unique prevalence of OSAS in this population [78,79,80,81]. A typical example of this would be the clinical picture of Down syndrome.

#### 1.1.4. Neuromuscular Diseases

OSAS is more common in children with neuromuscular diseases because of muscle hypotonia, which is frequently brought on by scoliosis, restrictive dysventilatoria syndrome, and muscle pump deficiency. These children exhibit dysventilatorial or atelectasic areas more frequently because of their lack of cough and relative incapacity to clear respiratory secretions. These variables favor the appearance of changes in gas exchanges by changing the ventilation/perfusion ratio.

#### 1.1.5. Hypercapnia

An elevated blood carbon dioxide level is among the signs and symptoms associated with hypercapnia. 

Abnormalities in the heart or lungs, such as respiratory acidosis or altered acid-base balance, are often the cause of this phenomenon. The inadequate ventilation of the alveoli is another common cause. Children are generally more likely than adults to suffer from hypercapnia when they sleep.

#### 1.1.6. Pediatric OSAS Symptoms and Signs

The most common symptoms of OSAS include chronic and persistent snoring (HS), often with breathing pauses, paradoxical or otherwise difficult night breathing, sleeping disorders with frequent night awakenings, excessive night sweating, and occasionally secondary enuresis (in a child who has acquired urinary continence for at least 6 months). Additional indications and symptoms at night include nightmares, agitation, adopting specific sleeping positions (such as saluting Mohammed), and a posture that causes the neck to extend excessively. Children with OSAS may exhibit signs and symptoms during the day, including excessive daytime sleepiness, headaches upon waking, irritability, and poor academic performance. There are occasionally opposing expressions in the two more traditional phenotypes. The adenotonsillar phenotype is often characterized by thinness and inadequate growth in addition to facies adenoidea. The issue in the obese phenotype is the opposite and is typified by overgrowth (Table 2).

### 1.2. Complications

There are three methods by which OSAS complications are assessed:

Arousals, or micro-awakenings, at the conclusion of hypnotic episodes; sporadic hypoxias with fast re-oxygenation (following the outlet at the end of apneic episodes), linked or unrelated to hypercapnia; and changes in intrathoracic pressure during obstructive events caused by respiratory effort.

These three processes function by initiating an intricate web of oxidative stress, free radical and pro-inflammatory cytokine release, elevated phlogosis indexes, epithelial dysfunction, and sympathetic nervous system activation. The result is decreased vagal tone; catecholamine release; and elevated heart rate and variability. Neurocognitive and behavioral issues, growth retardation, systemic arterial hypertension, pulmonary hypertension, and disorders of the cardiovascular and metabolic systems are all encouraged by this intricate network (Table 3).

### 1.3. Management

#### 1.3.1. Adenotonsillectomy (AT)

With an estimated 70–100% case efficacy, adenotonsillary hypertrophy is still the most frequent cause of OSAS in children, and the suggested course of treatment is still AT. A polysomnographic check will be scheduled following AT to determine whether any OSAS is still present.

#### 1.3.2. CPAP

Nasal continuous positive airway pressure, or CPAP, is a successful treatment for OSAS even in younger children. However, a major barrier to the efficient use of CPAP can be treatment adherence. Because of this, when AT is a more sensible option, it is not recommended to use CPAP as the first line of treatment for OSAS. However, CPAP is recommended for children who do not react well to surgery, children for whom surgery is not recommended, and children whose families refuse to give their consent for surgery.

#### 1.3.3. Medical Therapy

Numerous investigations have evaluated the effectiveness of leukotriene antagonists, such as montelukast, and topical nasal corticosteroids, such as fluticasone and budesonide, in the treatment of pediatric OSAS. Topical nasal corticosteroids are helpful for mild OSAS, but they should not be the only treatment for moderate or severe OSAS.

#### 1.3.4. Bariatric Surgery

In 2012, guidelines were released regarding the use of bariatric surgery to treat severe obesity in carefully selected adolescents. Numerous studies have demonstrated how well gastric bandages, gastric bypasses, and gastrectomy sleeves work to lower apnea indices (AHIs) and body mass indices (BMIs).

#### 1.3.5. Orthodontic Treatment

RME, or rapid maxillary expansion, has been used as a treatment for pediatric OSAS. The goal of this orthodontic procedure is to increase the hard palate’s transverse diameter by reopening the mid-palatal suture. This is accomplished by using a stationary apparatus with an expansion screw for approximately three to four months. Children with maxillary contraction and dental malocclusion typically undergo such a procedure.

## 2. Materials and Methods

### 2.1. Protocol and Registration

This systematic review was conducted following Preferred Reporting Items for Systematic Reviews and Meta-Analysis (PRISMA), and it was submitted to PROSPERO with number ID 490431.

### 2.2. Search Processing

We performed a search of databases such as Scopus, Web of Science (WoS), and PubMed using the keywords “OSA(S)” and “orthodontics” to select papers suitable for this topic, and the search was related to the last ten years (December 2013–December 2023).

### 2.3. Eligibility Criteria

The reviewers, in a double-blind manner, included papers that satisfied the following criteria for inclusion: (1) articles related to OSA(S) children undergoing an orthodontic treatment; (2) clinical studies or case series.

Exclusion criteria were represented by reviews (systematic and/or narrative) with/without meta-analyses, studies regarding adult populations, animal models, and in vitro studies.

### 2.4. Data Processing

The screening procedure, carried out by reading the article titles and abstracts chosen in the earlier identification step, allowed for the exclusion of any publications that varied from the themes looked at, and the full texts of publications previously included were then read. The reviewers discussed the selected articles, and in cases of disagreement, a third reviewer (FI) was consulted.

### 2.5. Quality Assessment

The quality of the included papers was assessed by two reviewers, R.F. and E.I., using ROBINS, a tool developed to assess the risk of bias in the results of non-randomized studies that compare the health effects of two or more interventions. Seven points were evaluated, and each was assigned a degree of bias. A third reviewer (F.I.) was consulted in the event of a disagreement until an agreement was reached.

## 3. Results

Keyword searches of the Web of Science (40), Scopus (11), and PubMed (705) databases yielded a total of 756 articles.

The subsequent elimination of duplicates (42) resulted in the inclusion of 714 articles.

Of these 714 studies, 665 were excluded because they deviated from the previously defined inclusion criteria (383 off-topic, 2 vitro/animal studies, 122 reviews, 135 adult studies, 23 no free full-text).

The screening phase ended with the selection of 16 publications for this work.

**Figure 1:**
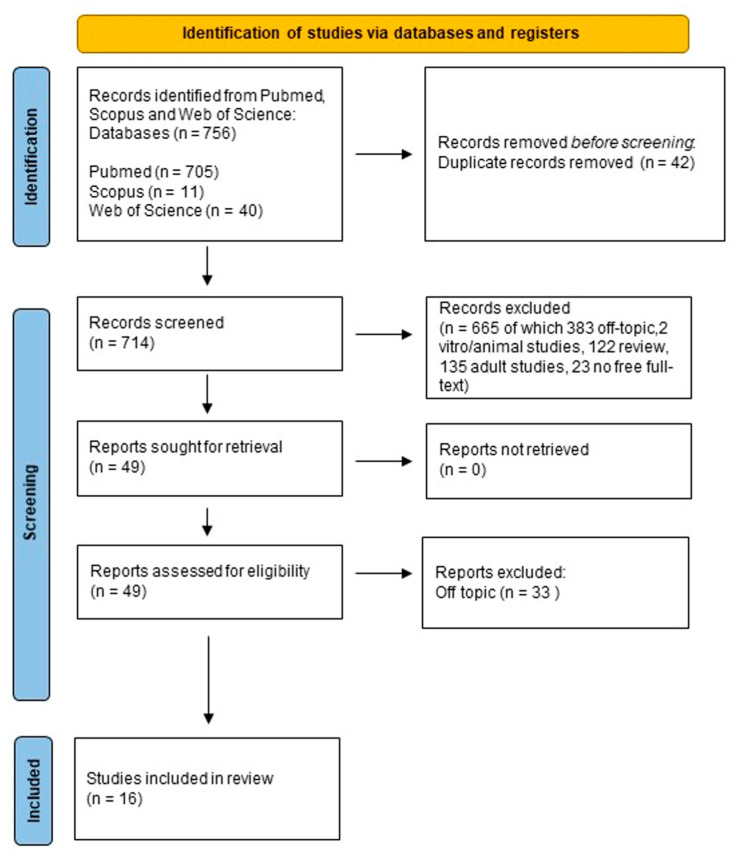
PRISMA flowchart used in this review paper.

The PRISMA flowchart of this review is summarized in Figure 1, and the data from each selected study (author(s), type of study, aim of the study, materials, and results) are reported in Table 4.

### Quality Assessment and Risk of Bias

**Scheme 1:**
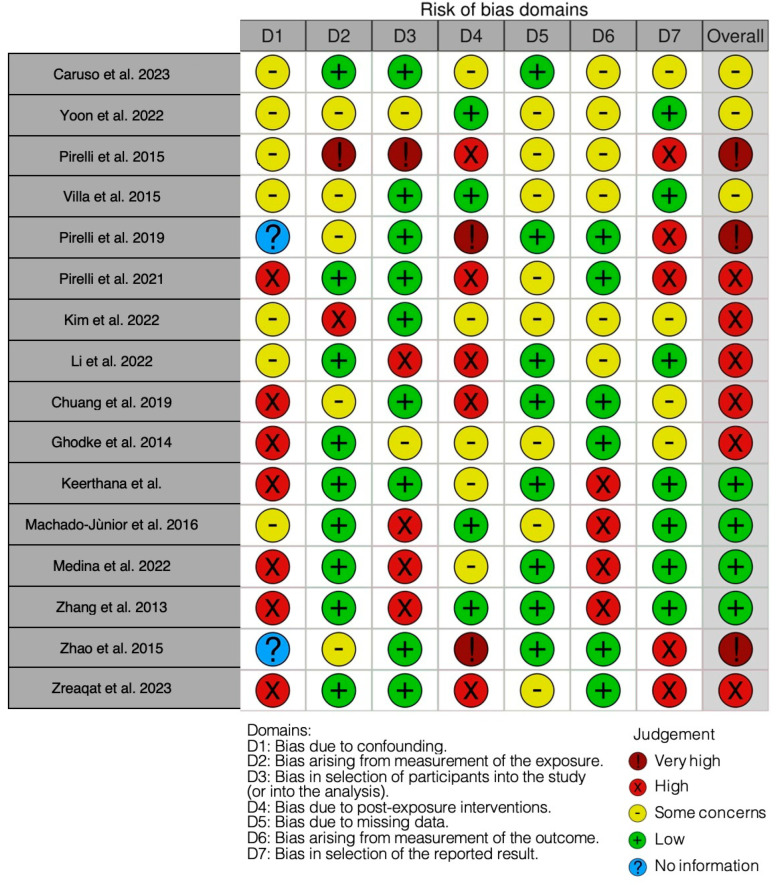
Bias assestment [82,83,84,85,86,87,88,89,90,91,92,93,94,95,96,97].

The risk of bias in the included studies is reported in Scheme 1. Regarding bias due to confounding, most studies have a high risk. The bias arising from measurement is a parameter with a low risk of bias. The majority of studies have a low risk of bias because of bias in the selection of participants. Bias due to post-exposure cannot be calculated because of high heterogeneity. Bias due to missing data is low in the majority of studies. Bias arising from the measurement of the outcome is low. Bias in the selection of the reported results is high in the majority of studies. The final results show that four studies have a low risk of bias, six studies have a high risk of bias, three have a very high risk of bias, and the remainder have a questionable risk of bias.

## 4. Discussion

### 4.1. OSAS Treatment with Rapid Palatal Expander

#### 4.1.1. Effectiveness of RPE in Modifying the Upper Airway

A 2023 clinical study by Caruso et al. evaluated cephalometric changes recorded in 14 young patients affected by class III malocclusion and OSAS treated with RPE and Delaire’s mask. In the cephalometric analysis, carried out on pre- and post-treatment latero-lateral radiographs, in addition to the classic values for verticality and sagittality, millimeter measurements of upper airway space dimensions were examined. At the end of therapy, there was a statistically significant increase in linear upper airway measurements and oropharyngeal and nasopharyngeal dimensions in all patients, creating an improvement in airway patency and OSAS-related clinical conditions [82].

A 2016 retrospective study by Yoon et al. evaluated the effectiveness of REP in decreasing palatine tonsil and adenoid volume in pediatric patients with OSA. Sixty children with an average age of 8 years were split into a study group treated with REP and a control group that received no treatment. At the end of therapy, patients treated with REP showed a statistically significant reduction in the volume of tonsils and adenoids in contrast to patients in the control group, in whom there was no improvement. This work is very interesting because it represents the first clinical study to quantify changes in tonsil volume following palatal expansion [83].

**Figure 2:**
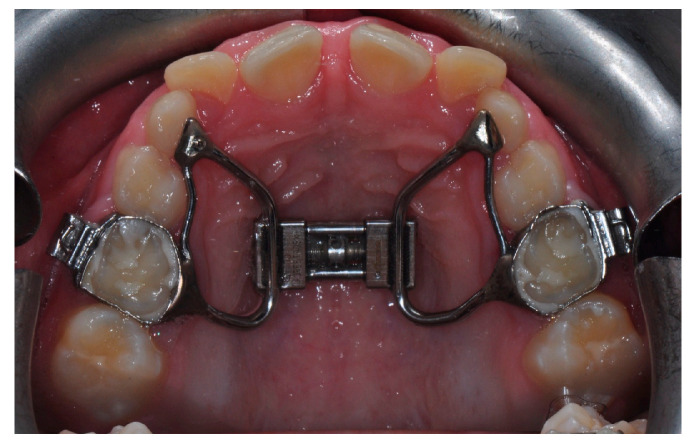
RPE device.

Kim et al., in a 2022 clinical study, assessed changes in respiratory function related to increased upper airway volume in patients with OSA treated with RPE. In all 26 cases treated, there was an increase in the size of the nasomaxillary complex, with improvement in parameters related to OSA: there was a reduction in AHI and oxygen saturation values, and snoring had markedly improved [88] (Figure 2).

#### 4.1.2. Radiographic Evaluation of the Effects of RPE

Pirelli et al., in a 2019 clinical study, evaluated the skeletal effects of RPE therapy in children with OSA through low-dose computed tomography (CT) measurements of the first molar angulation, maxillary base width, nasal cavity width, and the mid-palatal suture opening. The examinations performed demonstrated effective mid-palatal suture opening in all treated cases and improvements in the other parameters considered [86]. The same group of authors, in a 2021 study, demonstrated that RPE treatment is effective in children who have OSA and persistent snoring, causing an increase in the volume of the nasal cavity and nasopharynx. An increase in maxillary size results in an increase in upper airway volume, improving nasal breathing. The findings demonstrate that RPE therapy can eliminate obstructive sleep-breathing disorders and restore and enhance normal nasal airflow. The 19 children included in the study underwent CBCT before and after RPE treatment, and orthodontic and otolaryngological examination to confirm the above results [87].

#### 4.1.3. Alternative Treatments in Cases of RPE Failure

A 2022 clinical study by Li et al. investigated the effects of skeletally fixed trans-palatal distraction (TPD) on nasomaxillary expansion in patients with OSA previously treated with RPE. These patients, although they had resolved their malocclusions, still had residual OSA. As a result of this additional treatment, apnea episodes were significantly reduced: a nearly parallel anteroposterior opening of the mid-palatal suture enables the enlargement of the entire nasal passage with improved airflow characteristics in the nasal and pharyngeal airways. Improved airflow characteristics significantly correlated with enhanced polysomnographic results, indicating that nasomaxillary expansion is a feasible therapeutic option for patients who have previously undergone expansion [89].

#### 4.1.4. Long-Term Effectiveness

A 2015 clinical study by Pirelli et al. followed a group of 31 pediatric patients diagnosed with OSA treated with RPE over time. The patients, at the start of treatment, presented maxillary contraction in the absence of tonsillar and adenoid hypertrophy. At the end of treatment, annual follow-up was performed for the next 12 years. A total of 23 individuals completed follow-up and underwent final PSG. All patients showed stable orthodontic outcomes and resolution of OSA. RPE treatment, therefore, was also shown to be effective in the long term [84].

The same conclusions were reached in a 2015 retrospective clinical study by Villa et al., in which the benefits of RPE therapy in the resolution of malocclusions characterized by a high and narrow palate in patients with OSA and moderate tonsillar hypertrophy were evaluated. At the 10-year follow-up, most patients had resolved their OSA issues, and the best results were seen in those who underwent an orthodontic treatment earlier [85].

### 4.2. OSAS Treatment with Mandibular Advancement Devices

The condition known as obstructive sleep apnea (OSA) is characterized by the abnormal, intermittent total or partial blockage of breathing during sleep that interferes with regular ventilation. On the spectrum of obstructive breathing sleep disorders, it is the most severe kind [A]. Children with this kind of illness typically display symptoms throughout the day, such as irregularities in their behavior, development, cognitive abilities, and hearts [34].

Although the few data available now may imply that mandibular advancement appliances (MAAs) increase AHI scores, it is not possible to draw the conclusion that MAAs are useful in treating pediatric OSA. In 2016, in order to prove that evidence, Machado-Junior Almiro-Josè et al. conducted a randomized controlled pilot study.

They came to the conclusion that individuals who are hyperdivergent and undergoing thorough orthodontic treatment do not fare as well if they have OSA. Planning regular therapy for sleep-breathing problems and airway blockage should include an examination. The results of these children’s orthodontic therapy may be influenced by early detection and the treatment of pediatric OSA [93].

**Figure 3:**
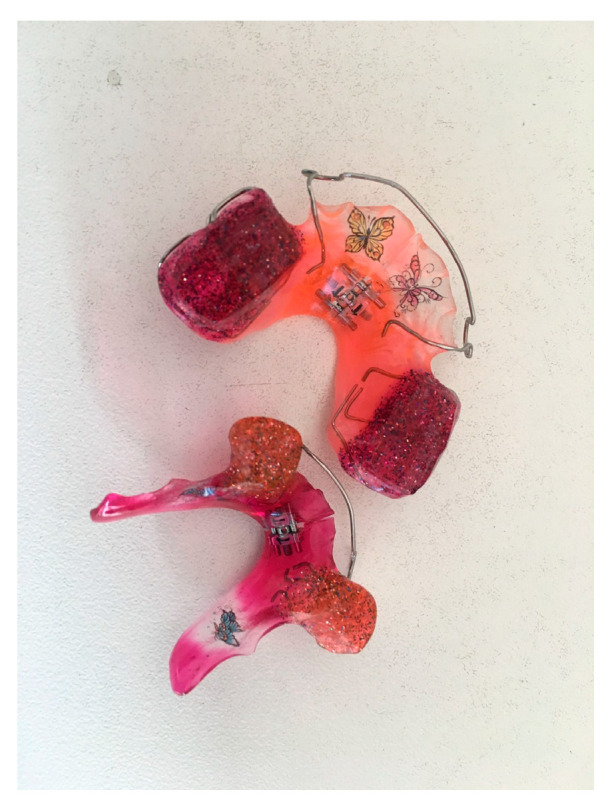
Twin block device.

One type of oral functional appliance used for the early treatment of children with mandibular retrognathia is called a twin block (TB) (Figure 3). Because of the mandible’s forward location, TB could be an appropriate oral appliance for treating children with OSA [98].

In a preliminary study conducted in 2013, Chen Z. et al. aimed to determine the initial effectiveness and tolerability of TB treatment for children patients with mandibular retrognathia and OSAS [9,99,100,101]. Taking into account certain limitations in the study’s design, such as the lack of a control group to compare with and the need to rule out other variables like growth that could have an impact on the findings, the authors came to the conclusion that TB appliances might help the selected patients with their facial profiles and OSA symptoms [95].

Ghodke S. et al., in a study conducted in 2014, analyzed the impact of TB appliance on the anatomy of pharyngeal airway passage (PAP) in a group of class II malocclusion patients in an age range of 8 to 14 years. They stated that, for class II malocclusion subjects, the TB appliance to treat mandibular retrusion enhanced PAP dimensions while maintaining the same pre-treatment posterior pharyngeal wall thickness. Consequentially, by removing predisposing factors and adaptive alterations in the upper airway during infancy, class II correction using a TB device may help lower the likelihood of developing OSA as an adult [91].

In a pilot study in 2018, Zhao T. et al. aimed to find out if OSA affects the orthodontic treatment outcome for class II hyperdivergent patients undergoing complete orthodontic therapy.

They highlighted how, in the young OSA patients selected for the study, the pattern of bone growth became more vertical, in contrast with the non-OSA group, where the pattern of bone growth became more horizontal. Along with that, both groups’ treatment outcomes in terms of occlusion and sagittal bone growth were comparable [96].

Keerthana P. et al., in 2022, presented a case series to highlight the effectiveness of an AdvanSync2 Class II corrector in the treatment of three orthodontic patients with OSA conditions. The modifications to airway size after the use of AdvanSync2 were evaluated by comparing lateral cephalograms taken before and after therapy. In all three cases, a notable improvement in airway dimensions was seen [92].

In a 2019 comparative cohort research, Chuang Li-Chuan et al. assessed the quality of life and craniofacial and airway morphology in children with OSA before and after a year of passive myofunctional therapy (PMFT). The PMFT device tested consistently in a custom-designed oral appliance with a built-in tongue bead. For the duration of the study (1 year) every night while they slept, study participants were to wear their appliances and roll the bead with their tongues.

They came to the conclusion that PMFT can enhance nasal breathing during sleep, as well as mandibular growth and upper airway morphology in the oropharyngeal region. The OSA-18 survey indicated a significant improvement in the quality of life of treated patients, particularly in relation to emotional distress [90].

In a 2023 study, Zreaqat M. et al. used cone beam computed tomography (CBCT) in conjunction with three-dimensional analysis to evaluate the effects of TB appliance therapy on upper airway parameters and dimensions, as well as the apnea–hypopnea indexes (AHIs), in children with OSA who had class II malocclusions and mandibular retrognathia before and after completing myofunctional TB therapy.

It has been demonstrated that CBCT imaging is a reliable and accurate diagnostic method for examining craniofacial tissues and upper airways [97].

The authors segmented the upper airway into three regions (nasopharynx, oropharynx, hypopharynx), and for each region, they measured the airway volume and the minimum cross-sectional area (MCA) in the axial view.

They concluded that the TB therapy performed to treat class II mandibular retrognathic skeletal malocclusion led to a significant decrease in AHI but no change in nasopharynx parameters. Upper airway volume; the MCA; the anteroposterior and lateral distances of the MCA at the level of the oropharynx; the MCA at the level of the hypopharynx; and upper airway length were significantly increased as a result of the findings [97].

Since, as we have shown, different studies agree that the mandible forward advancement with an orthodontic activator can improve the AHI in pediatric patients who have both abnormal maxilla–mandible relationships and OSA, Medina C.C. et al., in 2022, were interested in verifying the theory that, in addition to its intended function of inducing mandibular development, activators may enhance the dimensions of skeletal class II children’s upper airways to promote healthy sleep-breathing patterns even in the absence of sleep disturbances.

Many assessments, such as radiographic examinations of the upper airway, sleep-breathing monitoring, and questionnaires sent to parents and examined children, were used to test this theory.

They came to the conclusion that the activator not only allows for harmonic occlusion and healthy mandibular development but also widens the upper airway and lowers the frequency of disordered breathing events in children receiving this therapy, which enhances the quality of sleep and breathing [94].

## 5. Conclusions

Respiratory sleep disorders are a rather common condition in the pediatric population, OSAS being the most common among them. As in other fields of science, intervention as early as possible can change the natural course of the disorder. In this regard, an early orthodontic intervention, such as REP or mandibular advancement with functional appliances, may be effective in the management of pediatric OSAS, suggesting that the correction of craniofacial structure imbalances during growth can reduce snoring and OSAS in children and young adolescents. Specifically, there is limited evidence to support mandibular advancement appliances (MAAs) in improving pediatric OSA, so further investigation is needed to establish their efficacy conclusively, while studies that have evaluated RPE in pediatric patients with obstructive sleep apnea syndrome (OSAS) have shown promising results, with significant cephalometric changes finding an increase in linear upper airway measurements and the subsequent expansion of nasal airflow. The goal of this early interceptive treatment is clearly to restore the balance of the maxillary bone bases so that oral cavity functions (phonation, swallowing, breathing, and chewing) can be performed properly.

Further studies with a large number of patients are needed, especially on mandibular advancement devices used during the pediatric age, to evaluate their possible benefits in terms of OSAS-related symptoms and to develop structural modifications to improve airway morphology.

## Figures and Tables

**Table 1:** Some pathologies in which OSAS may be present [4].

Diseases with a Craniofacial Component	Associations with Soft Tissues	Neuromuscular Associations	Inflammatory Associations
Apert’s syndrome	Obesity	Cerebral palsy	Asthma
Crouzon’s syndrome	Cystic hygroma	Hypothyroidism	Metabolic syndrome
Pfeiffer’s syndrome	Papillomatosis (oroparingea)	Achondroplasia	Sickle cell disease
Pierre–Robin syndrome	Prader–Willi syndrome	Patients with cleft palate after repair	
Treacher Collins syndrome	Mucopolysaccharidosis	Down syndrome	
Goldenhar syndrome (hemifacial microsomy)	Beckwith–Wiedernann’s syndrome		
Atresia/coanal stenosis	Down syndrome		
Hallermann–Streiff’s syndrome	Syndromes with cleft		
Klippel–Feil syndrome			
Osteopetrosis			
Sickle cell anemia (sickle cell disease)			
Syndromes with cleft			

**Table 2:** Symptoms and signs of pediatric OSAS [3].

Night Symptoms	Daytime Speaker	Signs
Snoring	Difficulty awakening	Tonsil hypertrophy
Gasping	Lack of rest upon waking	High/wide lingual position
Noisy breathing (typically inspiratory)	Drowsiness	Growth disorders (obesity, insufficient growth)
Respiration paradox	Hyperactivity	Pulmonary hypertension
Indentations (jugular or rib)	Aggression, bad mood	Systemic hypertension
Apnee testifies	Oral respiration	Craniofacial abnormalities
Restless sleep	Meager appetite	Laryngeal
Hyperextension of the neck	Dysphagia	Obstruction of the nasal airways
Oral respiration	School difficulties	Hypotonia
Night sweating	Daytime speaker	Gastroesophageal reflux

**Table 3:** Complications of OSAS [2].

Metabolic	Neurocognitive	Cardiovascular
Increased C-reactive protein	Decreased quality of life	Autonomic dysfunction
Insulin resistance	Aggressive behavior	Systemic hypertension
Hypercholesterolemia	Poor school performance	Absence of drop in blood pressure in sleep
Increased transaminases	Depression	Left ventricular dysfunction
Reduced insulin-like growth factor	Attention deficit	Pulmonary hypertension
Reduced/altered growth hormone secretion	Hyperactivity	Variability of heart rate altered
	Moodiness	Increased vascular endothelial growth factor

**Table 4:** Examined articles.

Authors (Year)	Type of Study	Aim of the Study	Materials	Results
Caruso et al., 2023 [82]	Clinical study	To evaluate the cephalometric variations in upper airway dimensions and OSA-related clinical conditions after orthodontic treatment with an RME and the Delaire mask in patients affected by class III malocclusion.	In total, 14 children, ages 6 to 10, with mixed dentition were treated with a Delaire mask and an RME.	The study reports an increase in nasopharyngeal and oropharyngeal spaces. Improvements in OSAS-related clinical conditions and airway patency result from this increase.
Yoon et al., 2022 [83]	Retrospective cohort study	To assess the alterations in palatine and adenoid tonsil sizes subsequent to RPE through 3D volumetric analysis of cone beam computed tomography (CBCT) images.	In total, 60 pediatric patients with tonsillar hypertrophy were divided into 2 groups: one treated with RPE and a control group without any treatment.	RPE enormously decreased the size of the palatine and adenoid tonsils.
Pirelli et al., 2015 [84]	Clinical prospective study	To assess the long-term effectiveness of rapid maxillary expansion (RME) in a group of children with obstructive sleep apnea (OSA); RPE was found to significantly reduce the size of both the palatine and adenoid tonsils.	The study Ided 31 children with an OSA diagnosis. At baseline, these children had isolated maxillary narrowing and no enlarged adenotonsils. The subjects experienced repeated polysomnography (PSG) in their late teens or early 20s, as well as ongoing clinical evaluation.	Following RME treatment for a history of OSA, a subgroup of OSA children with isolated maxillary narrowing (initially followed up into adulthood) showed stable, long-term results.
Villa et al., 2015 [85]	Clinical retrospective study	To validate the effectiveness of RPE in children with mild to moderate adenotonsillar hypertrophy.	Pediatric patients with narrow palate, tonsillar hypertrophy, and OSA undergoing RPE therapy and reassessed over time.	RPE therapy was confirmed to be effective in resolving malocclusions associated with OSA.
Pirelli et al., 2019 [86]	Clinical prospective study	To evaluate the skeletal effects of RPE via low-dose computed tomography (CT).	In total, 14 pediatric patients with contracted palate and OSA underwent RPE therapy.	Opening of the mid-palatal suture was demonstrated in all cases.
Pirelli et al., 2021 [87]	Clinical study	To evaluate skeletal changes and changes in dimensions and volume of the upper airways before and after rapid RPE in children with OSA via CBCT. To evaluate if RPE therapy can improve both the patency of the nasal airways and the obstructive sleep apnea syndrome (OSAS).	In total, 19 pediatric patients with contracted palate and OSA underwent RPE therapy.	In all cases, the opening of the mid-palatal suture was demonstrated. Significant increases were observed in nasal osseous width, nasal cavity volume, total upper airway volume, and the nasopharynx and oropharynx.
Kim et al., 2022 [88]	Clinical study	To assess changes in respiratory function related to increased upper airway volume in patients with OSA treated with RPE.	In total, 26 patients (mean age, 13.6 years) were treated with RPE. Pre- and post-treatment CBCT assessment of airway and pre- and post-treatment assessment of mean apnea-hypopnea index (AHI).	In all cases, there was an increase in the size of the nasomaxillary complex, with improvement in parameters related to OSA.
Li et al., 2022 [89]	Clinical study	To evaluate the efficacy of nasomaxillary expansion via trans-palatal distraction (TPD) with skeletal anchorage in the resolution of OSA.	In total, 25 children without maxillary contraction with OSA were treated with TPD.	In children treated with RPE previously but without transverse maxillary deficiency, nasomaxillary expansion via skeletally anchored TPD improved OSA.
Chuang et al., 2019 [90]	Comparative cohort study	To compare the quality of life and the morphology of the airways, and cranium both before and after a year of passive myofunctional treatment (MFT) in children with OSA.	For a year, forty OSA youngsters who wore an oral device every night (the treatment group) and seventeen who did not (the control group) were monitored.	One year of passive MFT enhanced nasal breathing during sleep, as well as mandibular development and upper airway morphology in the oropharyngeal area.
Ghodke et al., 2014 [91]	Clinical study	To assess how class II malocclusion participants with retrognathic mandibles respond to a twin block device in terms of pharyngeal airway passage dimensions and posterior pharyngeal wall thickness.	Thirty-eight class II malocclusion individuals with mandibular retrusion, ages 8 to 14	The twin block device enhanced PAP dimensions while maintaining posterior pharyngeal wall thickness prior to treatment.
Keerthana et al., 2022 [92]	Case series	To summarize the results of using the AdvanSync2 Class II corrector in the treatment of three orthodontic patients who complained of breathing issues and a retrognathic mandible that interfered with their ability to sleep.	Three people who require treatment for class II malocclusion were linked to sleep apnea.	By increasing airway dimensions, the AdvanSync2 Class II corrector, when used in conjunction with fixed orthodontic equipment, improved the quality of life for class II patients.
Machado-Júnior et al., 2016 [93]	Pilot study	To assess mandibular advancement appliance in children who have OSA.	Adolescents diagnosed as apneic were those with an apnea–hypopnea index of one or more events per hour.	One year following the implementation of mandibular advancement devices, there was a reduction in the apnea–hypopnea index as compared with the non-user group.
Medina et al., 2022 [94]	Clinical study	To ascertain if using this equipment causes healthy children’s upper airways to enlarge and their sleep-breathing habits to improve.	In total, 39 healthy children: 20 for the activator group; 19 for the control group.	By opening the upper airway, the activator helps to enhance the quality of breathing during sleep.
Zhang et al., 2013 [95]	Clinical study	To look at how twin block (TB) appliances affect children who have mandibular retrognathia and OSA.	In total, 46 children (aged 9.7 ± 1.5 years, BMI: 18.1 ± 1.04 kg/m^2^) with mandibular retrognathia and OSA.	The patients’ facial profiles improved following therapy with the TB appliance.
Zhao et al., 2015 [96]	Pilot Study	To find out if class II hyperdivergent individuals undergoing complete orthodontic treatment are affected differently by the existence of OSA.	Patients who underwent orthodontic treatment and were between the ages of 12 and 14.	The results of these children’s orthodontic treatments may be impacted by the early detection and treatment of pediatric OSA.
Zreaqat et al., 2023 [97]	CBCT study	To assess rapid maxillary expansion (RME)’s long-term effectiveness in treating a group of children with obstructive sleep apnea (OSA).	In this study, 31 children with OSA diagnoses were included. At baseline, these children did not have enlarged adenotonsils and only had isolated maxillary constriction. The subjects experienced repeat polysomnography (PSG) in their late teens or early 20s, as well as ongoing clinical reevaluation.	Following RME therapy for juvenile OSA, a subgroup of children with isolated maxillary constriction who were first followed up into adulthood showed consistent, long-term improvements.

## Data Availability

Not applicable.

## Abbreviations

AT	Adenotonsillectomy
AHI	Apnea-hypopnea index
BMIs	Body mass indices
CBCT	Cone beam computational tomography
CPAP	Continuous positive airway pressure
CT	Computed tomography
MAA	Mandibular advancement appliance
MCA	Minimum cross-sectional area
OSA	Obstructive sleep apnea
OSAS	Obstructive Sleep Apnea Syndrome
PAP	Pharyngeal airway passage
PMFT	Passive myofunctional therapy
PSG	Polysomnography
RME	Rapid maxillary expansion
RPE	Rapid palatal expansion/Rapid palatal expander
TB	Twin Block
TPD	Nasomaxillary expansion using skeletally anchored trans-palatal distraction
